# Effect of Motion Graphic-Based Education on Knowledge, Practice and Recurrence of Hypoglycemia in Patients with Type 2 Diabetes: A Quasi-Experimental Study

**DOI:** 10.5812/ijem-166802

**Published:** 2026-01-31

**Authors:** Reyhaneh Azizi, Zohre Foroozanfar, Akram Mehrabbeik, Nasim Namiranian

**Affiliations:** 1 Diabetes Research Center, Non-communicable Diseases Research Institute, Shahid Sadoughi University of Medical Sciences, Yazd, Iran

**Keywords:** Diabetes Mellitus, Education, Hypoglycemia, Knowledge

## Abstract

**Background:**

Hypoglycemia is a common and serious complication in type 2 diabetes management, often leading to adverse health and social consequences. Education aimed at improving patient knowledge and self-care behaviors may prevent recurrence of hypoglycemic episodes.

**Objectives:**

This study investigated the effect of a motion graphics-based educational intervention on knowledge, practice, and recurrence of hypoglycemia among patients with type 2 diabetes in Yazd, Iran.

**Methods:**

In this quasi-experimental study, 122 patients with type 2 diabetes who referred to the Diabetes Center and had at least one hypoglycemic episode in the previous three months were randomly assigned to an intervention group (n = 60) or a control group (n = 62). The intervention group received standard care plus an educational video clip covering hypoglycemia definition, causes, symptoms, treatment, and prevention. Knowledge and practices (as primary outcomes) were assessed by validated questionnaires before and three months after the intervention, and the recurrence of hypoglycemia (as secondary outcome) was determined by patient self-report. Statistical analyses included McNemar’s test and logistic regression to compare within- and between-group differences.

**Results:**

Post-intervention, the intervention group showed significant improvements in knowledge about testing (from 76.7% to 91.7%, P = 0.022), treatment (61.7% to 90.0%, P = 0.001), and prevention (46.7% to 85.0%, P = 0.001) of hypoglycemia. Practice improvements included increased consultation with doctors (53.3% to 68.3%, P = 0.035) and implementation of preventive measures (41.7% to 66.7%, P = 0.001). Hypoglycemia recurrence decreased markedly from 100% to 35.0% (P = 0.001). Between-group comparisons confirmed statistically significant differences in key knowledge, practice, and hypoglycemia recurrence outcomes.

**Conclusions:**

Motion graphics educational content effectively enhanced hypoglycemia-related knowledge and preventive practices, leading to a significant reduction in hypoglycemic episodes among type 2 diabetic patients. Incorporating such interventions into diabetes care programs is recommended. It is worth noting that this study is limited by its reliance on self-reported hypoglycemia.

## 1. Background

Type 2 diabetes is a chronic condition that affects millions of people worldwide (1). According to the International Diabetes Federation (IDF) report, in 2024, the population of people aged 20 - 79 with diabetes worldwide was 588.7 million, which is expected to increase to 852.5 million by 2050 (2). Based on a meta-analysis study recently conducted in Iran, the estimated prevalence rate of diabetes was reported to be 13.40% between 2018 and 2023 (3). Although the primary goal of diabetes management is to maintain blood sugar in the normal range, strict control of blood sugar can sometimes increase the risk of hypoglycemia (4). Hypoglycemia, defined as serum glucose levels < 70 mg/dL (3.9 mmol/L), is one of the common complications associated with diabetes management, which can lead to serious consequences if not managed properly (5). Hypoglycemia can cause a wide range of problems, from job restrictions or insurance issues to disrupting patient adherence to treatment and increasing the risk of cardiovascular disease and related mortality (6, 7). Recurrent episodes of hypoglycemia can lead to a condition called hypoglycemia unawareness. In this state, individuals do not experience the usual symptoms that warn them of low blood sugar levels, which increases the risk of serious complications such as loss of consciousness or seizures (8). In addition, hypoglycemia-induced emotional and behavioral changes can also negatively affect the social life of people with diabetes and lead to interpersonal conflicts and arguments (9). Fortunately, episodes of hypoglycemia are largely preventable, and one approach in this regard is patient education (10). Considering that over 98% of diabetes management is conducted by patients and their caregivers, it is imperative for these individuals to possess adequate knowledge and competencies in this domain (11). Instructing individuals with diabetes regarding the signs, symptoms, risk factors, and preventive approaches is likely to yield favorable health-related outcomes (10, 12).

Since there are always obstacles in patient education such as time, access, and distance, using technology in education can be very helpful (13). According to the results of a meta-analysis, video-based interventions have a more favorable effect on health perceptions among low-literate individuals compared to traditional methods (14). Among the various electronic educational techniques, motion graphics warrant mention. A motion graphic constitutes a video or animation that generates an impression of movement or alters the visual characteristics of an element, often accompanied by auditory elements in digital media. The utilization of motion graphics as a medium is advantageous due to their capacity to recall emotional responses and facilitate the comprehension of information (15). According to our knowledge, in Iran, especially in Yazd, motion graphics have been used less in patient education. On the other hand, due to the frequency of hypoglycemia in patients with type 2 diabetes in Yazd (16) and previous successful experiences using technology in educating patients with diabetes (17, 18).

## 2. Objective

This study was designed to investigate the effect of motion graphic-based education on knowledge, practice, and recurrence of hypoglycemia in patients with type 2 diabetes.

## 3. Methods

### 3.1. Study Design and Participants

This quasi-experimental study was conducted on patients referred to the Diabetes Research Center, Yazd, Iran. Inclusion criteria comprised a diagnosis of type 2 diabetes for at least six months, age 18 - 65 years, at least one history of hypoglycemia in the past three months, no visual or hearing impairment, no cognitive problems, and access to a mobile phone to receive educational content. Participants who failed to receive and watch the educational clip despite follow-up were excluded from the study. The sample size was determined to be 60 people in each group using data from a similar study (19) with the aim of comparing the level of knowledge before and after the intervention, considering a type I error of 5%, a power of 80%, and a difference in the knowledge improvement of about 10.1% (the knowledge improvement in intervention group and control group 11.8% and 1.1% respectively). After selecting the sample, they were randomly divided into two intervention and control groups.

### 3.2. Measurement

The data for the present study were collected in two parts. The first part was data related to the demographic and clinical information of the patients, which was extracted from their medical records. For the second part of the information (patients' knowledge and practice regarding hypoglycemia), a questionnaire designed based on the standards of medical care in diabetes by the American Diabetes Association was used. The questionnaire included 5 questions, covering the definition, symptoms, testing, treatment, and prevention of hypoglycemia (20) (Appendix 1 in Supplementary File). The questionnaire was provided to 5 specialists (3 endocrinologists, 1 social medicine specialist, and 1 health education specialist) and approved. Also, 15 patients who were not part of the research sample approved the questions in terms of simplicity and comprehensibility.

A three-item checklist was used to assess patients' practice regarding hypoglycemia. The questions in this section were: “Do you check your blood sugar when you have signs and symptoms of hypoglycemia?”, “Have you consulted your doctor about hypoglycemia?”, “What steps have you taken to prevent hypoglycemia?”. For the first and second questions, the answer "Yes" was considered a correct answer, and regarding preventive measures, if patients mentioned at least two strategies they used to prevent hypoglycemia, it was considered a correct answer.

### 3.3. Intervention

After reviewing the literature, with emphasis on the latest clinical guidelines of the American Diabetes Association (ADA) (21), the dimensions of education and educational content in each dimension were developed. Educational content was prepared in five dimensions: Definition of hypoglycemia, cause, signs and symptoms, treatment methods, and prevention methods for hypoglycemia. The developed content was provided to a nutritionist and 4 endocrinologists to express their opinions on the accuracy and adequacy of the developed content. After making corrections and approving the educational content in a meeting consisting of endocrinologists, health education specialists, and a motion graphics team, the characteristics of the target group and the purpose of creating the clip were reviewed. In order to increase the chances of patients watching the clip in its entirety, an attempt was made to keep the duration of the clip short while maintaining the comprehensiveness of the content. The created clip was reviewed several times by experts until it reached the final approval stage (with a duration of 3′:57″) and then sent to the mobile phones of the participants in the intervention group via the internal messaging app "EETA". Those participants who did not have the desired application received the clip link via SMS. One week after sending, the participants were contacted and reminded to watch the clip. Also, a reminder SMS was sent to those who had not been able to watch the clip one week after the phone call. The intervention group received routine training. Three months after the intervention, the knowledge questionnaire and checklist of practice (as primary outcomes) were completed again for all participants and they were asked about the recurrence of hypoglycemia (as the secondary outcome).

### 3.4. Ethical Considerations

The present study was approved by the Ethics Committee of Shahid Sadoughi University of Medical Sciences (IR.SSU.REC.1400.256). The trial was registered at the Iranian Registry of Clinical Trials (IRCT20200426047208N3).

### 3.5. Statistical Analysis

Continuous variables were expressed as mean ± standard deviation, and qualitative variables were reported as number and percentage. In each group (intervention and control), changes in the correct answer before and after the intervention were analyzed using the McNemar test. Also, to compare the post-intervention outcomes between the two groups while adjusting for baseline values, logistic regression analysis was performed. Statistical analyses were performed using SPSS version 26. Statistical significance was set at a P-value < 0.05.

## 4. Results

### 4.1. Study Population and Baseline Characteristics

The study included 122 (intervention = 60 and control = 62) participants with a mean age of 55.26 ± 8.09 years. No significant difference was observed between groups regarding baseline characteristics. Other characteristics of participants in control and intervention groups are reported in Table 1. 

**Table 1.** Baseline Characteristics of Participants ^a^

Variables	Total	Intervention Group	Control Group	P-Value
**Age**	55.26 ± 8.09	54.91 ± 7.57	55.59 ± 8.61	0.654
**Age at diagnosis**	42.15 ± 8.21	42.25 ± 8.41	42.05 ± 8.07	0.893
**Duration of disease**	13.11 ± 7.98	12.66 ± 7.83	13.54 ± 8.16	0.544
**Gender**				0.616
Male	33 (27.0)	15 (25.0)	18 (29.0)	
Female	89 (73.0)	45 (75.0)	44 (71.0)	
**Education **				0.696
Illiterate	17 (13.9)	7 (11.7)	10 (16.1)	
Elementary	70 (57.4)	33 (55.0)	37 (59.7)	
Secondary	21 (17.2)	12 (20.0)	9 (14.5)	
Academic	14 (11.5)	8 (13.3)	6 (9.7)	
**Receiving insulin**	63 (51.6)	29 (48.3)	34 (54.8)	0.472
**HbA1c**	7.54 ± 1.26	7.55 ± 1.26	7.53 ± 1.27	0.944
**BMI **	29.89 ± 4.80	30.34 ± 5.08	29.46 ± 4.51	0.321
**History of education about hypoglycemia**	28 (23.0)	14 (23.3)	14 (22.6)	0.921

^a^ Values are expressed as mean ± SD or No. (%).

### 4.2. Knowledge, Practice, and Recurrence of Hypoglycemia

The results of knowledge, practice, and recurrence of hypoglycemia in intervention and control groups before and after intervention are reported in Table 2. 

**Table 2.** Knowledge, Practice, and Hypoglycemia Events ^a^

Questions	Total			Intervention Group			Control Group			P-Value ^b^
	Before	After	P-Value	Before	After	P-Value	Before	After	P-Value	
**Knowledge questions (correct answers) **										
Hypoglycemia definition	45 (36.9)	57 (46.7)	0.052	20 (33.3)	25 (41.7)	0.332	25 (40.3)	32 (51.6)	0.118	0.413
Hypoglycemia symptoms	83 (68.0)	90 (73.8)	0.265	42 (67.6)	49 (79.0)	0.118	41 (68.3)	41 (68.3)	1.00	0.132
Hypoglycemia testing	97 (79.5)	100 (82)	0.678	46 (76.7)	55 (91.7)	0.022	51 (82.3)	45 (72.6)	0.109	0.002
Hypoglycemia treatment	87 (71.3)	105 (86.1)	0.002	37 (61.7)	54 (90.0)	0.001	50 (80.6)	51 (82.3)	1.000	0.037
Hypoglycemia prevention	69 (56.6)	96 (78.7)	0.001	28 (46.7)	51 (85.0)	0.001	41 (66.1)	45 (72.6)	0.481	0.026
**Practice questions (yes)**										
Hypoglycemia testing	21 (17.2)	29 (23.8)	0.039	9 (15.0)	15 (25.0)	0.070	12 (19.4)	14 (22.6)	0.625	0.278
Consulting a doctor	64 (52.5)	77 (63.1)	0.031	32 (53.3)	41 (68.3)	0.035	32 (51.6)	36 (58.1)	0.454	0.215
Hypoglycemia prevention	55 (45.1)	70 (57.4)	0.037	25 (41.7)	40 (66.7)	0.001	30 (48.8)	30 (48.4)	0.100	0.020
**Hypoglycemia events (yes)**										
History of hypoglycemia in the past three months	122 (100)	54 (44.3)	0.001	60 (100)	21 (35.0)	0.001	62 (100)	33 (53.2)	0.001	0.044

^a^ Values are expressed as No. (%).

^b^ Comparison of response after training in two groups by adjusting the initial response.

#### 4.2.1. Knowledge Assessment Results

Hypoglycemia definition: The overall rate of correct responses improved modestly from 36.9% to 46.7% (P = 0.052), though this change did not reach statistical significance. Neither the intervention group (P =0.332) nor the control group (P = 0.118) showed significant individual improvements.

Symptoms of hypoglycemia: The percentage of patients who recognized at least two of the signs and symptoms of hypoglycemia increased from 67.6 to 79 in the intervention group after training, which was not statistically significant (P = 0.118). However, the control group did not show any improvement in this regard. Also, the difference between the groups was not significant (P = 0.132).

Hypoglycemia testing: When evaluating knowledge about the importance of testing blood glucose levels upon symptom onset, the intervention group exhibited significant enhancement from 76.7% to 91.7% (P = 0.022). Conversely, the control group experienced a decrease from 82.3% to 72.6% (P = 0.109). The comparison between groups revealed a statistically significant difference (P = 0.002).

Hypoglycemia treatment: Regarding knowledge of the appropriate hypoglycemia treatment, the intervention group showed marked progress from 61.7% to 90.0% (P = 0.001), while the control group maintained consistent performance at approximately 80%. The comparison between groups demonstrated statistical significance (P = 0.037).

Hypoglycemia prevention: Knowledge of hypoglycemia prevention measures improved dramatically in the intervention group from 46.7% to 85.0% (P = 0.001). The control group displayed minor improvement from 66.1% to 72.6% (P = 0.481). The between-group difference achieved statistical significance (P = 0.026).

#### 4.2.2. Practical Application Results

Hypoglycemia Testing: The practice of testing blood sugar during symptomatic episodes showed marginal overall improvement from 17.2% to 23.8% (P = 0.039). The intervention group progressed from 15.0% to 25.0% (P = 0.070), while control group changes were minimal. No significant inter-group difference emerged (P = 0.278).

Healthcare consultation behavior: Regarding seeking medical advice for hypoglycemia, the intervention group demonstrated significant improvement from 53.3% to 68.3% (P = 0.035), while the control group showed moderate progress. The between-group comparison did not reach significance (P = 0.215).

Hypoglycemia prevention: The intervention group exhibited considerable improvement in preventive action implementation from 41.7% to 66.7% (P = 0.001), while the control group remained relatively stable at approximately 48%. The inter-group analysis showed statistical significance (P = 0.020).

Hypoglycemia recurrence: A significant decrease in hypoglycemia occurrence was observed following the educational intervention. Initially, all participants (100%) reported experiencing hypoglycemic episodes, which declined to 44.3% overall post-training (P = 0.001). The intervention group demonstrated a more substantial reduction from 100% to 35.0% (P = 0.001) relative to the control group, which decreased from 100% to 53.2% (P = 0.001). The between-group analysis confirmed a significant difference (P = 0.044).

**Figure 1.**
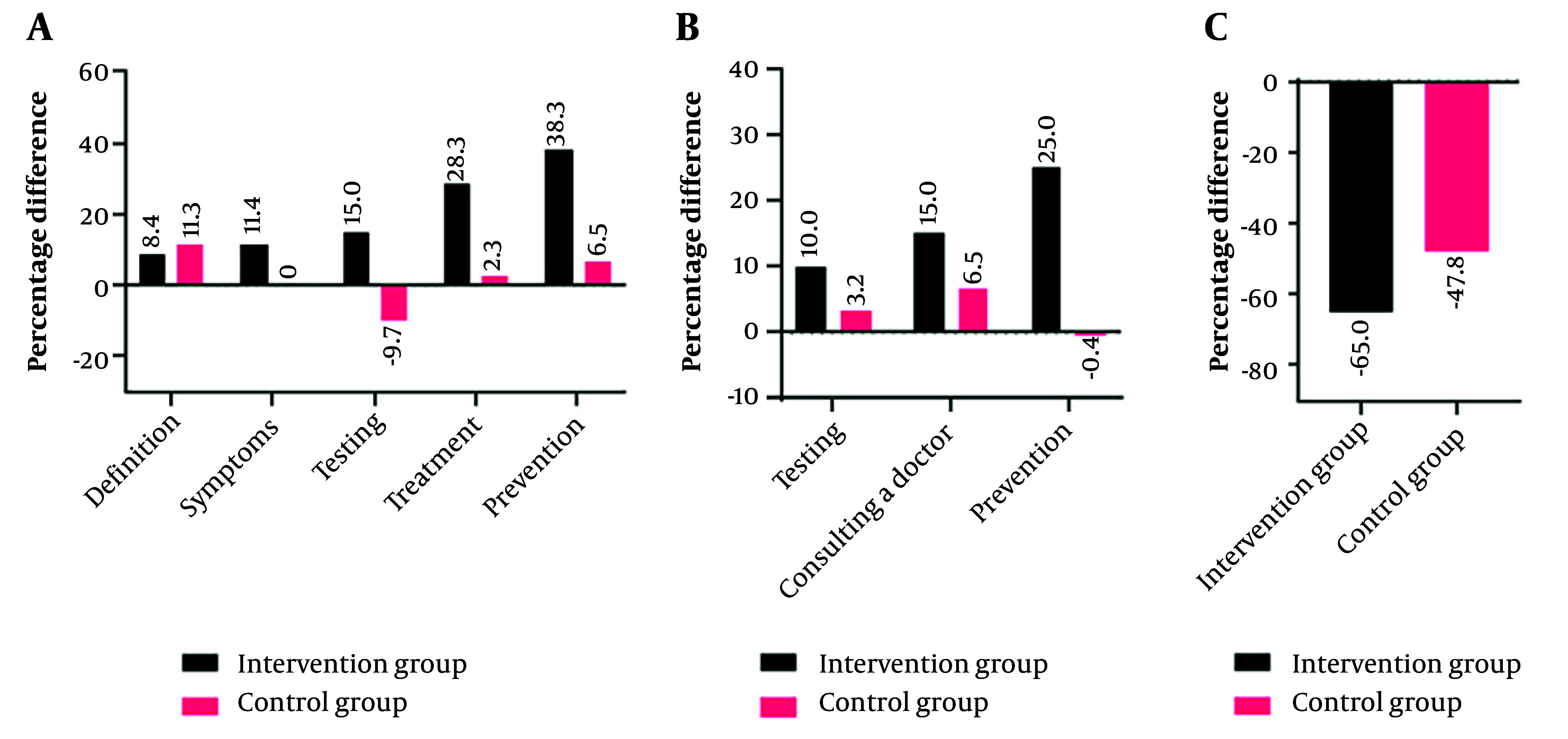
Percentage difference of knowledge (A), practice (B), and hypoglycemia events (C) before and after intervention

Percentage difference of knowledge (A), practice (B), and hypoglycemia events (C) before and after intervention in the intervention and control groups are reported in Figure 1. In all outcomes except for the definition of hypoglycemia, the difference in the percentage of correct responses and appropriate performance before and after the intervention was higher in the intervention group.

## 5. Discussion

In this study, we evaluated how video-based education impacted comprehending what hypoglycemia is and how to manage it among type 2 diabetic patients in Yazd, Iran. The findings demonstrate that after the education, in the intervention group, hypoglycemia-related knowledge and practice improved significantly compared to the control group. Also, the percentage of reported recurrence of hypoglycemia in the intervention group decreased significantly after education.

In terms of knowledge, the percentage of people with the correct definition of hypoglycemia increased in the intervention group after the education, although it was not statistically significant. This result is in contrast to the results of a study conducted by Chu et al. (20) and is debatable in several ways. According to the literature, the threshold for the onset of hypoglycemic symptoms in diabetic patients may be elevated due to poor glucose control and a history of hypoglycemia (10). Since the participants in this study all had a history of hypoglycemia, some of them may have experienced symptoms at glucose levels > 70 mg/dL and therefore, they consider a higher than actual value as hypoglycemia. Furthermore, considering that not all home glucose meters provide accurate readings at low blood glucose levels, in addition to teaching the scientific definition of hypoglycemia (serum glucose levels < 70 mg/dL), patients are reminded to always consider the possibility of a 10 - 15% error when interpreting the results, and therefore values greater than 70 are established in the minds of patients as the level of hypoglycemia (22).

Based on the result, the educational clip had a significant impact on increasing patients' knowledge about the necessity of hypoglycemia testing when signs and symptoms occur. This improvement holds substantial significance for diabetes management. Clinical experiences have demonstrated that some individuals with diabetes experience signs and symptoms of hypoglycemia at glucose levels above 70 mg/dL. These symptoms are frequently attributable to psychosomatic factors, including anxiety and sleep disturbances (23). Moreover, in people with poor glycemic control and no history of hypoglycemia, elements of the autonomic stress response, and corresponding symptomatology, may be elicited at higher plasma glucose concentrations. In such cases, hypoglycemia testing prevents unnecessary glucose consumption, which could compromise effective blood sugar control (24).

Knowledge of hypoglycemia prevention methods is a key part of managing this condition, which in the present study significantly increased in the intervention group. In line with the findings of this study, previous studies conducted by Eghtedari et al. (25) and Ratri et al. (26) have also shown the effect of video training (including motion graphics) on increasing the knowledge of patients with diabetes. In fact, the nature of such educational content allows patients to access and review the material at any time, thus retaining the material in their memory (27).

In terms of practice, the percentage of people who talked to their doctor about hypoglycemia increased from less than half before the intervention to about two-thirds after the intervention. This part of the findings is notable because, despite the guidelines' emphasis on screening and counseling for hypoglycemia at every visit, previous studies conducted in different parts of the world indicated that hypoglycemia is not properly addressed during visits (12, 28, 29). Considering that information seeking regarding disease prevention and treatment is low in developing countries (30), the present study suggests that educational programs can encourage patients to engage more actively with healthcare providers, which is a critical component of effective diabetes management (31).

Based on the results, after the educational intervention, the percentage of patients who took preventive strategies against hypoglycemia increased significantly in the intervention group. The positive effect of using video-based training in improving the practice of patients with type 2 diabetes was confirmed in previous similar studies (18, 32). These findings emphasize the beneficial impact of educational interventions on enhancing patients’ awareness of early detection, which is essential for preventing severe hypoglycemic episodes (33).

Although people's knowledge about the necessity of hypoglycemia testing increased, their performance in this area did not show significant improvement. Financial constraints and lack of insurance coverage for medical equipment have always been one of the obstacles to diabetes management (18, 34). In the present study, the cost of strips and glucometers could be a possible reason for not testing blood sugar when hypoglycemic symptoms occur.

According to the patients' self-report, the recurrence of hypoglycemia was reduced at the end of the study in both groups, but this reduction was significantly greater in the intervention group than in the control group. The reduction in hypoglycemia in the control group can be attributed to the relative increase in patients' knowledge following routine hypoglycemia care, which naturally affects the incidence of hypoglycemia (35), and the difference between the two groups reflects the high effectiveness of structured education in improving self-care skills and preventing diabetes-related complications, which has been repeatedly demonstrated (21, 36).

The use of motion graphics-based education, as a new and engaging method, was a strength of our study, as it increased attention and effectiveness of education. However, the reliance on patients' self-reports regarding the recurrence of hypoglycemia can be considered a limitation of this study, and it would have been better to use a continuous monitoring device.

In conclusion, teaching key points about hypoglycemia in the form of a short but comprehensive motion graphic clip can increase knowledge and improve practice, and consequently reduce the recurrence of hypoglycemia in patients with type 2 diabetes. Therefore, it is recommended that healthcare providers use such training to empower patients to better manage hypoglycemia.

ijem-24-1-166802-s001.pdf

## Data Availability

The dataset presented in the study is available on request from the corresponding author during submission or after publication.

## Clinical Trial Registration Code:

IRCT20200426047208N3.

## References

[1] Mehrabbeik A, Tofighi D, Khalilzadeh SH, Azizi R, Namiranian N, Azad F (2024). Factors Associated with Achieving HbA1c Targets in Patients with Type 2 Diabetes.. Jundishapur J Chronic Dis Care..

[2] Magliano DJ, Boyko EJ (2022). IDF diabetes atlas..

[3] Hazar N, Jokar M, Namavari N, Hosseini S, Rahmanian V (2024). An updated systematic review and Meta-analysis of the prevalence of type 2 diabetes in Iran, 1996-2023.. Front Public Health..

[4] Al-Azayzih A, Kanaan RJ, Altawalbeh SM, Alzoubi KH, Kharaba Z, Jarab A (2024). Prevalence and predictors of hypoglycemia in older outpatients with type 2 diabetes mellitus.. PLoS One..

[5] American Diabetes Association (2021). 6. Glycemic Targets: Standards of Medical Care in Diabetes-2021.. Diabetes Care..

[6] Johnson-Rabbett B, Seaquist ER (2019). Hypoglycemia in diabetes: The dark side of diabetes treatment. A patient-centered review.. J Diabetes..

[7] Salih JM (2024). Glycemic Profiles and Hypoglycemia Awareness Among Pregnant Women with Gestational and Pre-existing Diabetes Referred to a Tertiary Center in Sulaimaniyah-Iraq in 2024.. Int J Endocrinol Metab..

[8] Przezak A, Bielka W, Moleda P (2022). Fear of hypoglycemia-An underestimated problem.. Brain Behav..

[9] Hermanns N, Heinemann L, Freckmann G, Waldenmaier D, Ehrmann D (2019). Impact of CGM on the Management of Hypoglycemia Problems: Overview and Secondary Analysis of the HypoDE Study.. J Diabetes Sci Technol..

[10] Nakhleh A, Shehadeh N (2021). Hypoglycemia in diabetes: An update on pathophysiology, treatment, and prevention.. World J Diabetes..

[11] Rahbar S, Zarifsanaiey N, Mehrabi M (2024). The effectiveness of social media-based microlearning in improving knowledge, self-efficacy, and self-care behaviors among adult patients with type 2 diabetes: an educational intervention.. BMC Endocr Disord..

[12] Bukhsh A, Goh BH, Zimbudzi E, Lo C, Zoungas S, Chan KG (2020). Type 2 Diabetes Patients' Perspectives, Experiences, and Barriers Toward Diabetes-Related Self-Care: A Qualitative Study From Pakistan.. Front Endocrinol..

[13] Cochran J (2023). The Development of Video-Based Diabetes Education for Integration in the Primary Care Setting [Thesis]..

[14] Galmarini E, Marciano L, Schulz PJ (2024). The effectiveness of visual-based interventions on health literacy in health care: a systematic review and meta-analysis.. BMC Health Serv Res..

[15] Yaakob TKST, Azahari NA, Abdullah S, Manaf ARA, Mahamood AF, Yusoff NIKM (2021). Use of interactive video based on motion graphics to create awareness on handling stress.. Proceedings of Green Design and Manufacture 2020..

[16] Shabestari M, Mehrabbeik A, Barbieri S, Marques-Vidal P, Heshmati-Nasab P, Azizi R (2025). Predictive factors of hypoglycemia in type 2 diabetes: a prospective study using machine learning.. Sci Rep..

[17] Salarkarimi F, Karandish M, Zakerkish M, Abbaspoor Z (2024). Impact of WhatsApp-Based Self-Care Education on Self-Care Behaviors and Lifestyle in Overweight and Obese Pregnant Women with Diabetes: A Randomized Controlled Trial.. Jundishapur J Chronic Dis Care..

[18] Mehrabbeik A, Azizi R, Namiranian N (2023). [Effect of Insulin Injection Re-Education on Reducing Injection Errors in Patients with Type 2 Diabetes].. J Shahid Sadoughi Univ Med Sci..

[19] Tok Czen A, Ozcan S (2024). The effects of a hypoglycaemia education programme on the outcomes in patients with type 2 diabetes.. Kontakt..

[20] Chu LT, Nguyen TQ, Pham PTT, Thai TT (2021). The Effectiveness of Health Education in Improving Knowledge about Hypoglycemia and Insulin Pen Use among Outpatients with Type 2 Diabetes Mellitus at a Primary Care Hospital in Vietnam.. J Diabetes Res..

[21] American Diabetes Association (2022). Standards of Care in Diabetes-2023 Abridged for Primary Care Providers.. Clin Diabetes..

[22] Sonmez A, Yilmaz Z, Uckaya G, Kilic S, Tapan S, Taslipinar A (2010). The accuracy of home glucose meters in hypoglycemia.. Diabetes Technol Ther..

[23] Divilly P, Martine-Edith G, Zaremba N, Soholm U, Mahmoudi Z, Cigler M (2024). Relationship Between Sensor-Detected Hypoglycemia and Patient-Reported Hypoglycemia in People With Type 1 and Insulin-Treated Type 2 Diabetes: The Hypo-METRICS Study.. Diabetes Care..

[24] Amiel SA (2021). The consequences of hypoglycaemia.. Diabetologia..

[25] Eghtedari M, Goodarzi-Khoigani M, Shahshahani MS, Javadzade H, Abazari P (2023). Is Web-Based Program Effective on Self-Care Behaviors and Glycated Hemoglobin in Patients with Type 2 Diabetes: A Randomized Controlled Trial.. Iran J Nurs Midwifery Res..

[26] Ratri DMN, Hamidah KF, Puspitasari AD, Farid M (2020). Video-based health education to support insulin therapy in diabetes mellitus patients.. J Public Health Res..

[27] Hoe CYW, Ahmad B, Watterson J (2024). The use of videos for diabetes patient education: A systematic review.. Diabetes Metab Res Rev..

[28] Pilla SJ, Park J, Schwartz JL, Albert MC, Ephraim PL, Boulware LE (2021). Hypoglycemia Communication in Primary Care Visits for Patients with Diabetes.. J Gen Intern Med..

[29] Jeon JH (2018). Letter: Patient Understanding of Hypoglycemia in Tertiary Referral Centers (Diabetes Metab J 2018;42:43-52).. Diabetes Metab J..

[30] Mengiste M, Ahmed MH, Bogale A, Yilma T (2021). Information-Seeking Behavior and Its Associated Factors Among Patients with Diabetes in a Resource-Limited Country: A Cross-Sectional Study.. Diabetes Metab Syndr Obes..

[31] Chrvala CA, Sherr D, Lipman RD (2016). Diabetes self-management education for adults with type 2 diabetes mellitus: A systematic review of the effect on glycemic control.. Patient Educ Couns..

[32] Damayantie N, Ernawati E, Dewi M (2025). Impact of the Health Belief Model on Hypoglycemia Prevention Skills in Diabetes Mellitus Patients.. Health Educ Health Promot..

[33] Davies MJ, D'Alessio DA, Fradkin J, Kernan WN, Mathieu C, Mingrone G (2018). Management of Hyperglycemia in Type 2 Diabetes, 2018. A Consensus Report by the American Diabetes Association (ADA) and the European Association for the Study of Diabetes (EASD).. Diabetes Care..

[34] Brohi AH, Hakim A, Wassan SM, Soomro AM, Bhatti WS, Tunio AH (2020). Facilitators and barriers to self-monitoring of blood glucose (SMBG) in diabetic patients.. J Pharm Res Int..

[35] Bakar A, Qomariah SN, Santoso CH, Gustomi MP, Syaful Y, Fatmawa L (2020). Factors the incidence of hypoglycemia in diabetes mellitus patients: A pilot study in the emergency room.. Enferm Clin..

[36] Powers MA, Bardsley JK, Cypress M, Funnell MM, Harms D, Hess-Fischl A (2020). Diabetes Self-management Education and Support in Adults With Type 2 Diabetes: A Consensus Report of the American Diabetes Association, the Association of Diabetes Care & Education Specialists, the Academy of Nutrition and Dietetics, the American Academy of Family Physicians, the American Academy of PAs, the American Association of Nurse Practitioners, and the American Pharmacists Association.. J Am Pharm Assoc..

