# Supplementary material for: Effect of Motion Graphic-Based Education on Knowledge, Practice and Recurrence of Hypoglycemia in Patients with Type 2 Diabetes: A Quasi-Experimental Study
Source: Int J Endocrinol Metab. 2026 Jan 31;24(1):e166802. doi: 10.5812/ijem-166802 (PMC13187686; doi:10.5812/ijem-166802)
Supplement: ijem-24-1-166802-s001.pdf [file ijem-24-1-166802-s001.pdf]

Question	Score
1. What blood sugar level is defined as hypoglycemia?<br>□ 1.Below3.9 mmol/L (or 70 mg/dL)<br>□ 2.Below 11.1 mmol/L (or 200 mg/dL.)<br>□ 3.Below 7.0 mmol/L (or 126 mg/dL)<br>□ 4. Others<br>□ 5. Do not know	1 point for answering "Below 3.9 mmol/L (or 70 mg/dL)"
2. What is the main symptoms of hypoglycemia? (Multiple choice question)<br>□ 1.Sweating<br>□ 2.Rapid pulse<br>□ 3.Weakness, fatigue<br>□ 4. Dizziness<br>□ 5. Headache<br>□ 6. Hungry, appetite<br>□ 7. Blurred vision<br>□ 8. Anxiety, agitation<br>□ 9. Tremble<br>□ 10. Irritability<br>□ 11. Do not know	1 point for answering at least two symptoms
3. When you have suspicious symptoms of hypoglycemia, is it necessary to test your blood sugar level?<br>□ 1.Yes<br>□ 2. No<br>□ 3. Do not know	1 point for answering "Yes"
4.What are proper treatment for hypoglycemia? (Multiple choice question)<br>□ 1.Eat or drink 15 grams of fast-acting sugar (glucose tablets, sugary candy, soft drink, fruit juice, sweets)<br>□ 2.Recheck blood sugar levels 15 minutes after using fast-acting sugar<br>□ 3.If blood sugar level is still low, repeat eating or drinking fast-acting sugar<br>□ 4. Eat or drink short-acting sugar (bread, pyramidal rice dumpling, low-fat milk, one meal per day)<br>□ 5. Do not know	1 point for answering at least "Eat or drink 15 grams of fast-acting sugar (glucos tablets, sugary candy, soft drink, fruit juice, sweets)"
5. What are effective ways to prevent hypoglycemia? (Multiple choice question)<br>□ 1.Do not eat late/skip meals<br>□ 2.Check blood sugar level regularly<br>□ 3.Take medication/insulin with right time and right dose<br>□ 4. Ensure that insulin dose is consistent with meals<br>□ 5. Always bring fast-acting sugar<br>□ 6. Light meal before intense activities<br>□ 7. Restrict alcohol intake. Do not drink without eating<br>□ 8. Bring blood sugar monitoring chart and report on the episodes of hypoglycemia to your doctor in the follow-ups<br>□ 9. Do not know	1 point for answering at least two symptoms
Total point range	0-5

Appendix 1. Knowledge about hypoglycemia
